# Investigation of parent-of-origin effect in comitant strabismus using MOD score analysis

**Published:** 2009-06-09

**Authors:** Sherin Shaaban, Toshihiko Matsuo, Konstantin Strauch, Hiroshi Ohtsuki

**Affiliations:** 1 Department of Ophthalmology, Okayama University Graduate School of Medicine, Dentistry, and Pharmaceutical Sciences, Okayama City, Japan; 2 Department of Ophthalmology, Mansoura Ophthalmic Center, Mansoura University, El-Mansoura City, Egypt; 3 Institute of Medical Biometry and Epidemiology, Philipps University Marburg, Marburg, Germany

## Abstract

**Purpose:**

Comitant strabismus is a common pediatric ophthalmic disorder with both genetic and non-genetic factors contributing to its etiology. The aim of the current study is to investigate the phenomenon of a parent-of-origin effect, genomic imprinting, as a possible mode of inheritance in comitant strabismus.

**Methods:**

We performed parametric genome-wide MOD score (model-maximized LOD score) linkage analysis, incorporating imprinting effects, for 382 microsatellite markers in a sample of 258 individuals (117 males and 141 females) from 55 Japanese families with comitant strabismus. We included individuals as affected patients if they presented with comitant esotropia or exotropia based on ophthalmic examination, history taking, or analysis of medical records.

**Results:**

Significant or suggestive linkage to comitant strabismus with evidence of maternal or paternal imprinting was detected at D4S1575 (4q28.3), D7S486 (7q31.2), D11S1320 (11q24.2), D12S324 (12q24.32), and D19S420 (19q13.11). Using the MOD score approach, we found new evidence of linkage to comitant strabismus at three loci on chromosomes 6q26 (MOD_imp_=MOD_reg_=3.75), 12q24.32 (MOD_imp_=3.36), and 19q13.11 (MOD_imp_=3.79).

**Conclusions:**

The results suggest that the parent-of-origin effect may play a role in the etiology of comitant strabismus.

## Introduction

Comitant strabismus is a common ophthalmic disorder. It is the misalignment of the eyes where the angle of deviation is constant in all directions of gaze, which is unlike the incomitant type where the angle is variable. Comitant strabismus accounts for 95% of the cases of strabismus. It includes several clinical subtypes such as esotropia, exotropia, hypertropia, microtropia, and monofixation syndrome. A strong genetic background for its etiology has been suggested by twin, family, and population studies. Comitant strabismus is thought to be inherited as a complex genetic trait [1-5]. Genome-wide linkage analysis of comitant strabismus so far has only yielded few chromosomal susceptibility loci [6,7].

Identifying the genes for a complex trait such as comitant strabismus might be hindered by factors such as incomplete penetrance, phenocopies, genetic heterogeneity, and polygenic inheritance [8]. With these factors in mind, choosing a proper inheritance model together with a suitable method of linkage analysis is of paramount importance. One approach for adequately modeling a complex trait is to perform parametric linkage analysis under a few simple models. One limitation of this approach is that a misspecification of the disease-model parameters can reduce the power to detect linkage and in cases of multi-marker analysis, can even exclude linkage [9]. Another approach is to maximize the LOD score over the trait-model parameters, thus performing a MOD score analysis [10], which is also known as maximizing the maximum LOD score (MMLS) [11]. This approach can be a more powerful method to detect linkage, albeit at the cost of increasing type I errors [12].

The phenomenon of genomic imprinting is another dimension worthy of investigation in a complex disorder such as comitant strabismus. Genomic imprinting or parent-of-origin effect is a mechanism by which only one copy of a gene pair is expressed. This expression is determined by the parental origin of the copy [13,14]. This functionally haploid state eliminates the protection that diploidy normally confers against the deleterious effects of recessive mutations. Moreover, the expression of imprinted genes can be deregulated epigenetically. Thus, imprinted genes represent susceptibility loci that can be functionally altered by both genetic and epigenetic events. Although many chromosomal regions in the human genome are likely to be imprinted, particularly those involved in development [15,16], imprinting is not accounted for in the usual linkage analysis. Incorporating information on imprinting may improve the power to detect linkage if the locus of interest is in fact imprinted [17].

In our earlier work to investigate chromosomal susceptibility loci for comitant strabismus, we performed non-parametric linkage analysis (NPL) with parametric analysis and opted to choose only two simple models of inheritance (recessive and dominant) for the parametric analysis [7,18]. In the current study, we re-examined our linkage data set of families with comitant strabismus by a rather more exploratory method (MOD score analysis) [10,11,19,20] to investigate the parent-of-origin effect as a possible mode of inheritance and to seek new chromosomal susceptibility loci for comitant strabismus.

## Methods

### Study sample and phenotype

The study population included 258 individuals from 55 Japanese families mainly residing in Okayama region of Japan. Forty-seven families were nuclear and eight were extended pedigrees. Those families were previously recruited for a genetic study of comitant strabismus [7]. There were 117 males (45%) and 141 females (55%) in this study. Each family had at least two members affected with comitant strabismus. All adults or parents of children participating in the study gave informed consent. The protocol was approved by the Ethics Committee of Okayama University Medical School (Okayama City, Japan) and adhered to the tenets of the Declaration of Helsinki.

Phenotypic ascertainment was based on the results of the ophthalmological examination of probands and/or their available relatives, family history, and medical records data analysis. We included any proband or available relative as affected if they presented constant or intermittent esotropic, exotropic, or hypertropic comitant misalignment in the form of heterotropia (manifest misalignment) or heterophoria (latent misalignment). Any unavailable relative with a history of strabismus and/or strabismic amblyopia, strabismus surgery, or wearing spectacles to correct strabismus was also considered affected. The demographic data of the participating families and data validation methods are described in details elsewhere [7].

### Genotyping

Three hundred eighty-two polymorphic microsatellite markers were typed for 214 individuals on autosomal chromosomes 1-22 with an average spacing of 10 cM. The genotyping technique was previously reported [7,18].

### Linkage analysis

We used GENEHUNTER-MODSCORE software [21,22] to perform a multipoint linkage analysis for our data set. GENEHUNTER-MODSCORE is an extension of GENEHUNTER software version 2.1 [23-25] and GENEHUNTER-IMPRINTING [26]. This program allows for maximization of parametric LOD scores over the parameters of the proposed trait model, i.e., the penetrances (ƒ) and disease allele frequency (P). Furthermore, it can take genetic imprinting in account when calculating the parametric multipoint LOD scores.

Two modeling options were chosen for the analysis, one allowing for imprinting (calculating MOD_imp_ score) and one without (calculating MOD_reg_ score). When non-imprinting was chosen, the trait model consisted of the disease allele frequency and three penetrance parameters [ƒ_(+/+)_, ƒ_(Het),_ and ƒ_(m/m)_] where “+” denotes the wild-type allele and “m” denotes the mutation. “ƒ_(Het)_” denotes the penetrance for individuals who are heterozygous at the disease locus irrespective of the parental origin of the mutation. For the imprinting model, we specified four penetrance parameters [ƒ_(+/+)_,ƒ_(m/+),_ ƒ_(+/m)_, and ƒ_(m/m)_], assuming that the first allele is derived from the father and the second allele is derived from the mother. Hence, GENEHUNTER-MODSCORE treats paternal and maternal transmission of the disease allele in a different way. If the heterozygote penetrance values, ƒ_(m/+)_ and ƒ_(+/m)_, in the best fitting model proposed by the program were different, genomic imprinting was indicated and an imprinting index (I) was calculated as follows:

$$\text{I}=\frac{f(\text{m/+})-f(\text{+/m})}{f(\text{m/m})-f(\text{+/+})}$$

I=1.0 when maternal imprinting is complete, I=−1.0 when paternal imprinting is complete, and nonzero values in between represent partial genomic imprinting [27].

The option “modcalc single” was used for the analysis. This allows for a separate maximization over trait models for each assumed disease locus position. The “penetrance restriction” and “allfreq restriction” options were activated, and “dimensions” was set to “2” (i.e., default values). When the “penetrance restriction” is set to “on”, this means that the heterozygote penetrance parameters (two of them in case of imprinting) are constrained to be not smaller than the homozygous wild-type penetrance, ƒ_(+/+)_, and not greater than the homozygous mutant penetrance, ƒ_(m/m)_. On the other hand, activating the “allfreq restriction” option means that the disease allele frequency is constrained to be not greater than the value specified by the “highest allfreq” command (which defaults to 0.5). The “dimensions” option refers to the number of parameters that are jointly varied during the fine maximization of a MOD score analysis. Since there is no evidence that prevalence of comitant strabismus differs between the two sexes, we used the same penetrance parameters for males and females. Marker allele frequencies were based on counting alleles in all individuals within the data set by the computer program DOWNFREQ version 1.1 (1995) [28,29]. Map locations were taken from the 1996 GENETHON human linkage genetic map (Kosambi distances in cM) [30].

To determine significance in the interpretation of results, we applied the following guidelines. In standard parametric linkage analysis, a LOD score of 3.0, which gives an asymptotic p value of 0.0001, is considered a significant linkage finding [31]. In the case of MOD score analysis, the resulting LOD scores are usually inflated due to maximization over several parameters, which results in an increase in type I errors. To correct for this, an adjustment in the range of 0.3-1.0 should be applied to the resulting MOD scores [12,32].

On the other hand, to conclude if imprinting is present at a certain locus or not, we examined both the imprinting index values at that locus and the difference between MOD scores obtained under the imprinting and the non-imprinting models. It is proposed that one can infer the correct mode of inheritance (MOI) by investigating the differences between LOD scores calculated under different models. With a difference of 1.5 between two MOIs, the superior LOD score reflects with high reliability the correct MOI while a difference of 2.5 or more practically guarantees correct inference of MOI [26,33].

## Results

**Figure 1:**
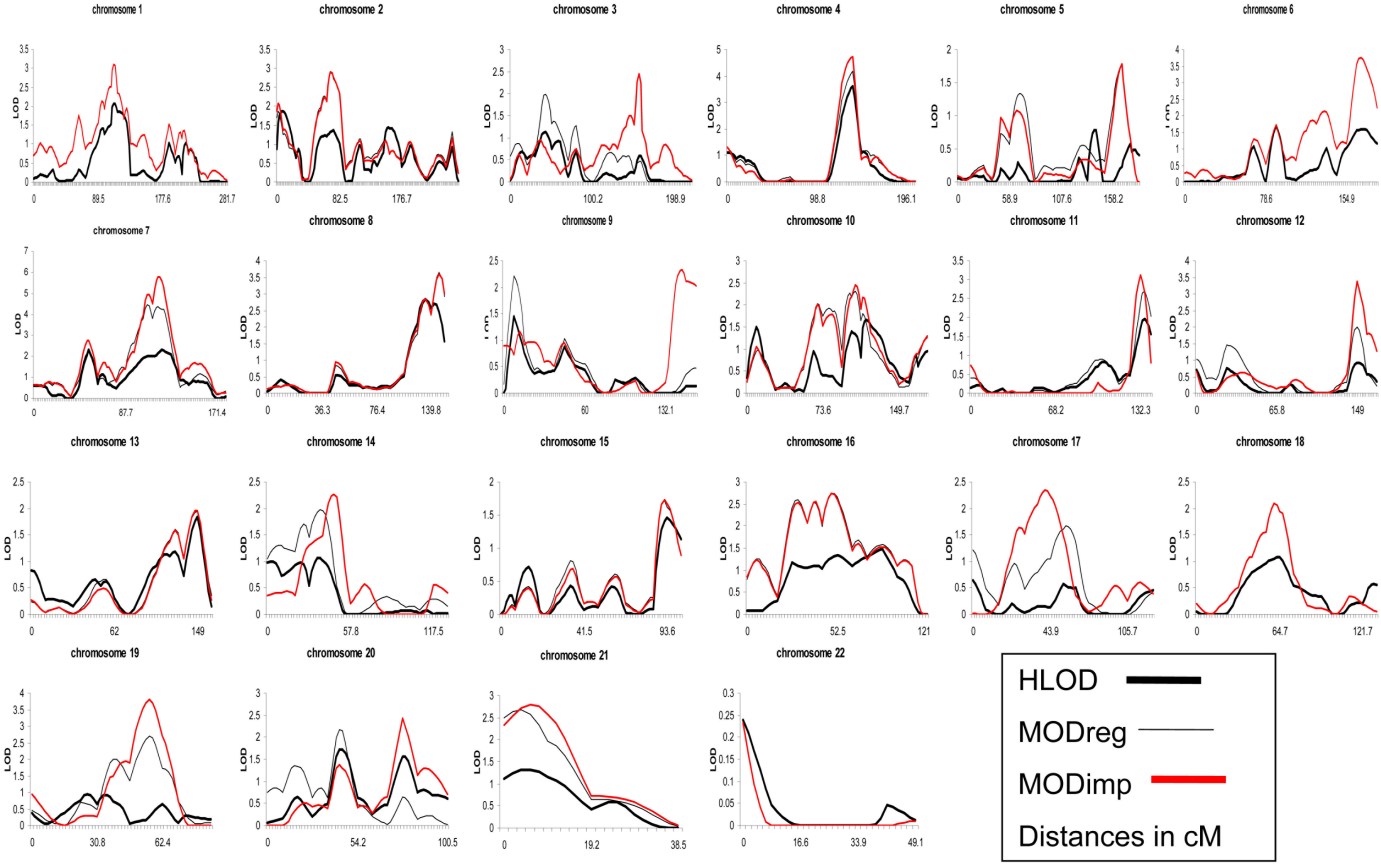
HLOD and MOD scores for the 22 autosomal chromosomes. Parametric genome-wide linkage analysis results of the autosomal chromosomes in 55 Japanese families with comitant strabismus.  Solid thick black lines=Heterogeneity logarithm of the odds “HLOD”; solid thin black lines=MOD scores for the non-imprinting model “MODreg”, and solid thick red lines=MOD scores for the imprinting model “MODimp”. For each chromosome, the HLOD scores displayed are reported for either the dominant or the recessive model (whichever shows the overall highest score) with the assumption of disease allele frequency (P_m_) being 0.01, penetrance being 0.8, and a phenocopy rate for the non-gene carriers being 0.01 [7]. MOD_imp_ and MOD_reg_ curves for chromosomes 1, 6, 18, and 22 are identical.

Figure 1 represents plots of the genome-wide multipoint linkage analysis at the 22 autosomal chromosomes. The results are represented in the form of HLOD_,_ MOD_reg_, and MOD_imp_ scores_._ HLOD is the heterogeneity logarithm of the odds calculated under two simple models of inheritance, dominant or recessive (detailed results of HLOD scores were previously reported) [7]. MOD_reg_ is the model-maximized LOD score assuming a non-imprinting model with only three penetrance parameters, and MOD_imp_ is the model-maximized LOD score assuming an imprinting model with four penetrance parameters.

Table 1 shows the genetic regions for which MOD score calculation leads to a disease model with ƒ_(m/+)_ not equaling ƒ_(+/m)_, which indicates a parent-of-origin effect (genomic imprinting), together with the best fitting parameters for the trait model at these regions. The non-imprinting results are also shown in Table 1. In most of those genetic regions, the maximum MOD_imp_ and MOD_reg_ scores are reported at the same markers. On a few occasions, the maximum scores are reported for adjacent markers if the loci yielding the maximum MOD_imp_ and MOD_reg_ scores are separated by an interval of less than 10 cM.

**Table 1:** MOD scores and estimated trait model parameters at loci suggestive of imprinting.

**Chromosome**	**Adjacent marker and position in cM**	**MOD**	**Penetrances**					**P_(m)­_**
			**ƒ_(+/+)_**	**ƒ_(m/+)_**	**ƒ_(Het)_**	**ƒ_(+/m)_**	**ƒ_(m/m)_**	
2	D2S337							
	73.10 (imprinting model)	2.90	0.01	0.025		0.01	0.3	0.11
	73.10 (non-imprinting model)	2.87	0.008		0.015		0.25	0.11
4	D4S1575							
	130.60 (imprinting model)	4.68	0.00	0.46		0.29	0.46	0.000001
	130.60 (non-imprinting model)	4.15	0.00		0.4		0.4	0.003
7	D7S486							
	120.24 (imprinting model)	5.78	0.005	0.09		0.005	1.0	0.02
	120.24 (non-imprinting model)	4.35	0.01		0.01		0.53	0.05
8	D8S284							
	146.88 (imprinting model)	3.59	0.002	0.08		0.07	1.0	0.004
	146.88 (non-imprinting model)	3.54	0.001		0.05		1.0	0.002
10	D10S1686							
	111.86 (imprinting model)	2.45	0.008	0.008		0.04	0.37	0.05
	111.86 (non-imprinting model)	2.31	0.008		0.02		0.32	0.06
11	D11S1320							
	132.30 (imprinting model)	3.12	0.002	0.14		0.002	1.0	0.0006
	135.54 (non-imprinting model)	2.68	0.0001		0.6		0.6	0.00001
12	D12S324							
	149.00 (imprinting model)	3.36	0.003	0.06		0.006	0.06	0.015
	149.00 (non-imprinting model)	1.99	0.0008		0.0008		0.11	0.01
14	D14S276							
	45.58 (imprinting model)	2.26	0.008	0.008		0.12	0.12	0.015
	D14S288							
	37.72 (non-imprinting model)	1.96	0.03		0.03		0.4	0.15
17	D17S921							
	40.62 (imprinting model)	2.34	0.00	0.49		0.00	0.49	0.4
	D17S1857							
	49.84 (non-imprinting model)	1.55	0.06		0.06		0.63	0.35
19	D19S420							
	57.04 (imprinting model)	3.79	0.0008	0.0008		0.015	0.04	0.045
	57.04 (non-imprinting model)	2.69	0		0.0001		0.0001	0.08
21	D21S1256							
	5.16 (imprinting model)	2.79	0.002	0.05		0.03	1.0	0.003
	3.44 (non-imprinting model)	2.66	0.002		0.02		0.43	0.01

cM stands for centiMorgan. MOD scores for the imprinting or the non-imprinting models were obtained at the same or nearby markers. “+” means wild-type allele, and “m” denotes mutant allele with the paternally inherited allele reported first. P_(m)_ is the disease allele frequency. The results are reported for the loci where the heterozygote penetrance ƒ_(m/+)_ was unequal to the heterozygote penetrance ƒ_(+/m)_ indicating genomic imprinting.

Tendency to maternal imprinting [paternal expression; ƒ_(m/+)_>ƒ_(+/m)_] was found at several loci. The strongest evidence was found near D4S1575, D11S1320, D12S324, and D17S921. The best fitting model at D17S921 denoted complete maternal imprinting (I=1.0). The difference between the imprinting versus the non-imprinting models was 0.79, and the MOD score at that locus was rather low (MOD_imp_=2.34).

The MOD_imp_ scores at chromosomes 4, 11, and 12 were more than 3.0. Maternal imprinting was near complete at chromosome 12 (I=0.95). MOD_imp_ at D12S324 was 3.36, and the difference between the imprinting and the non-imprinting models was as large as 1.37; while partial maternal imprinting was observed on chromosomes 4 and 11 (Imprinting index=0.37 and 0.14, respectively). The difference between MOD_imp_ and MOD_reg_ at D4S1575 was 0.53, and the difference at D11S1320 was 0.44.

Tendency to paternal imprinting [maternal expression; ƒ_(m/+)_<ƒ_(+/m)_] was obtained at chromosomes 10, 14 and 19. MOD_imp_ at D19S420 was 3.79, and the best fitting model at that locus indicated partial paternal imprinting (I=-0.36) with a noticeable difference between the imprinting and the non-imprinting MOD scores being 1.1. Complete paternal imprinting was observed at chromosome 14 (I=-1.0), yet the difference between the imprinting and the non-imprinting MOD scores was 0.3 and MOD_imp_ was only 2.26.

The most prominent linkage peak was observed at 120.24 cM on chromosome 7 (MOD_imp_=5.78). The difference between MOD_imp_ and MOD_reg_ at this locus was as large as 1.43. However, the heterozygote penetrances of the best fitting model differed only slightly (I=0.09).

The difference between MOD_imp_ and MOD_reg_ and that between the heterozygote penetrances at the remaining loci, displayed in Table 1, were indicative of partial genomic imprinting.

In addition to the results of genomic imprinting, we observed new evidence of linkage to comitant strabismus at 6q26, 12q24.32, and 19q13.11 (Figure 1, Table 2). The MOD score at D6S264 was 3.75 with no difference between MOD_reg_ and MOD_imp_ at this locus. The MOD_imp_ score at D12S324 was 3.36 while the MOD_imp_ score at D19S420 was 3.79. The 1-LOD interval at the three loci extended from 166.50 cM to 184.26 cM on chromosome 6, from 143.68 cM to 154.46 cM on chromosome 12, and from 52.88 cM to 62.40 cM on chromosome 19. No evidence of linkage at these loci could be obtained in our previous work calculating HLOD scores under the dominant or recessive models [7].

**Table 2:** MOD scores for the imprinting (MODimp) and the non-imprinting (MODreg) models at chromosomes 6, 12 and 19 together with the non-parametric linkage analysis scores (NPL).

**Chromosome**	**Adjacent marker**	**cytogenetic location**	**position of maximum LOD (cM)**	**1-LOD CI (cM)**	**MOD_imp_**	**MOD_reg_**	**NPL**	**p value**
6	D6S264	6q26	172.32	17.76	3.75	3.75	2.59	0.002
12	D12S324	12q24.32	149.0	10.78	3.36	1.99	2.0	0.01
19	D19S420	19q13.11	57.04	9.52	3.79	2.69	2.75	0.001

cM means centiMorgan, 1-LOD CI stands for 1-LOD confidence interval in centiMorgans, and NPL stands for non-parametric linkage score. The p value is the value for NPL scores. The MOD scores at the three loci show new evidence of linkage to comitant strabismus which was not previously reported in the same data set [7].

Table 3 is a summary of the HLOD, MOD_reg_, and MOD_imp_ scores together with the non-parametric linkage score (NPL) and its p value for all the loci that have been previously reported to show evidence of linkage to comitant strabismus by us and by others [6,7]. As shown in Table 3 and Figure 1, MOD_reg_ and/or MOD_imp_ scores at any of these loci were larger than the HLOD scores obtained previously in the standard parametric and non-parametric linkage analyses. A noticeable increase in the MOD scores compared to the HLOD score was observed for the loci at 1p31.3, 4q28.3, 7p14.3, 7q31.2, 8q24.13, and 11q24.2.

**Table 3:** MOD scores at all the loci previously reported to show evidence of linkage to comitant strabismus.

**Chromosome**	**Adjacent marker**	**HLOD**	**MOD_reg_**	**MOD_imp_**	**NPL**	**p value**
1p31.3	D1S207	2.07	3.10	3.10	1.96	0.01
1q31.1	D1S413	2.10	1.97	2.30	2.19	0.008
4q28.3	D4S1575	3.62	4.15	4.68	2.68	0.001
7p*	D7S513	0.29	0.83	1.13	1.56	0.04
7p14.3	D7S484	2.32	3.05	3.27	2.32	0.005
7q31.2	D7S486	2.32	4.35	5.78	2.7	0.001
8q24.13	D8S284	2.83	3.54	3.59	2.28	0.006
11q24.2	D11S1320	1.97	2.68	3.12	1.87	0.02
20q11.23	D20S195	2.01	2.17	2.19	2.39	0.004

HLOD=the highest heterogeneity logarithm of the odds calculated under either a recessive or dominant model with a disease allele frequency (P) of 0.01 [7]. MOD_reg_ is the MOD score calculated for the non-imprinting model. MOD_imp_ is the MOD score calculated for the imprinting model. NPL means non-parametric linkage score. The asterisk points to a locus on 7p reported by Parikh et al. [6]; no evidence of linkage was obtained at the same locus in our data set [7].

## Discussion

The comitant form of strabismus is much more common than the incomitant form, yet more insight about the genetics underlying the inheritance of incomitant strabismus have been revealed [1,2]. To date, only three studies have addressed the inheritance in comitant strabismus by performing genome-wide linkage scans and revealing only few susceptibility loci [6,7,18].

We chose to analyze our set of data by the MOD score method, which is one of the most comprehensive ways in analyzing linkage data by providing information about evidence of linkage and trait locus position and giving an estimate of the disease model parameters [10,11,21]. We used GENEHUNTER-MODSCORE software, which allowed us to test for genomic imprinting as a possible mode of inheritance in our data set of families with comitant strabismus. To the best of our knowledge, no reports about genomic imprinting in comitant or incomitant forms of strabismus have yet been published.

Eleven loci showed evidence of genomic imprinting (Table 1) with differences between MOD_imp_ and MOD_reg_ ranging from 0.03 to 1.43 and with absolute values of the imprinting index (I) ranging between 0.02 to 1 (an absolute value of 1 refers to complete paternal or maternal imprinting). Out of the 11 loci where evidence of genomic imprinting was observed, seven loci showed a tendency to maternal imprinting. One of the most interesting findings was the locus on chromosome 12q24.32 (D12S324) with near complete maternal imprinting and a remarkable difference between the imprinting and the non-imprinting MOD scores. A maternally imprinted gene; Fibrosin-like 1 (*FBRSL1*); was reported by Leudi et al. [16] at a nearby locus on chromosome 12q24.33. Partial paternal imprinting was detected on chromosome 19q13.11 (D19S420). That locus contains a maternally imprinted gene; carbohydrate (N-acetylgalactosamine 4-0) sulfotransferase 8 (*CHST8*) and is next to a region containing a paternally imprinted gene coding for a putative uncharacterized protein LOC400692 (*Q8N3U1*) at 19q13.13 [16].

Complete paternal and maternal imprinting was obtained at D14S276 and D17S921, respectively. The smaller disease allele frequency in the imprinting model for D14S276 [P_(m)_=0.015] compared to the non-imprinting model [P_(m)_=0.15] is in favor of the imprinting model. This is due to the fact that in case of trait-model misspecification, a decrease in the LOD score can be avoided by specifying a larger disease allele frequency [9], and thus, an inflated estimate of the disease allele frequency obtained in a MOD score analysis may indicate an inappropriate modeling. On the other hand, there was only moderate differences between the MOD_imp_ and the MOD_reg_ scores at either of the loci on chromosomes 14 or 17, and the MOD score could at most be considered weakly suggestive of linkage to comitant strabismus.

**Figure 2:**
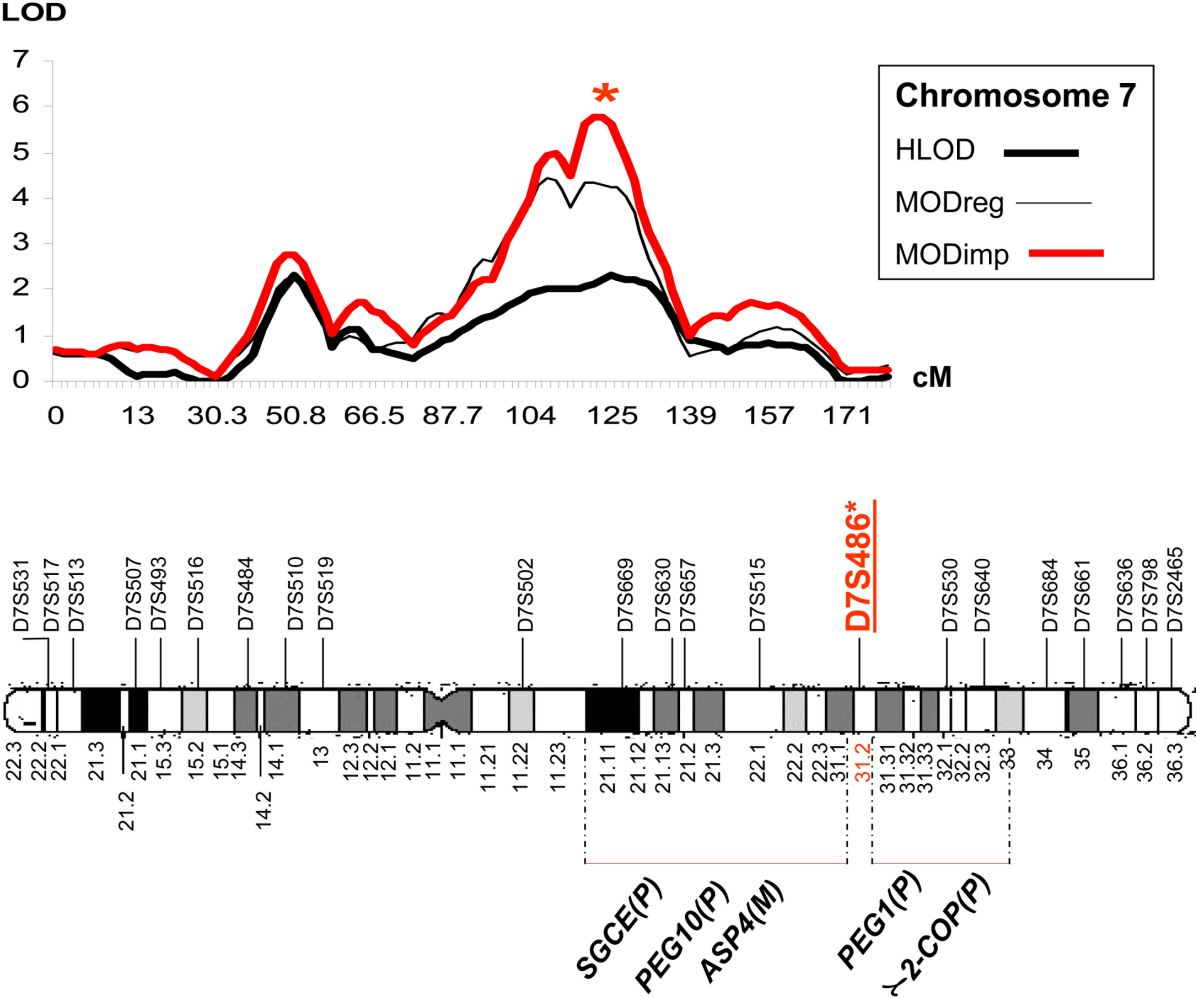
HLOD and MOD scores for chromosome 7. Above: Solid thick black lines=Heterogeneity logarithm of the odds (HLOD); solid thin black lines=MOD scores for the non-imprinting model “MOD_reg_” and solid thick red lines=MOD scores for the imprinting model “MOD_imp_”. HLOD scores are calculated under a recessive model with disease allele frequency P_m_=0.01, penetrance=0.8 and a phenocopy rate for the non-gene carriers=0.01 [7]. Below: A schematic representation of chromosome 7 (MOD_imp_=5.78) was obtained at 7q31.2 between two blocks of imprinted genes [34,35].  (P)=paternally imprinted gene, (M)=maternally imprinted gene.

The parent-of-origin effect on chromosome 7 is worthy of careful investigation. In a previous study, we reported significant evidence of linkage to comitant strabismus at 7q31.2 [7]. The MOD_imp_ score of 5.78 obtained in the current study at this locus was the highest among all loci. In addition, there was a significant difference between the imprinting and the non-imprinting MOD scores (1.43), albeit with a small difference between the heterozygote penetrances at this locus. Moreover, we observed that the homozygous penetrance (m/m) changed from reduced penetrance (0.53) in the non-imprinting model to complete penetrance (1.0) when imprinting was allowed. It can be speculated that the reduced penetrance in such a case is attributed to model misspecification when imprinting is not considered while the locus might actually be imprinted. The linkage at 7q31.2 is interesting because that locus lies directly between two regions that have been reported to have blocks of imprinted genes (Figure 2) [34,35]. Given its location between these two imprinted blocks and with a noticeable difference between the imprinting and the non-imprinting MOD scores, this locus should be a candidate for thorough investigation in future studies.

It is noteworthy that many of the first reported human genomic imprinting disorders such as Prader Willi syndrome, Angelman syndrome, and Wolf-Hirschhorn syndrome [14,36,37] share strabismus as a common characteristic. The incidence of strabismus in Angelman syndrome is about 42%, and the percentage increases to 66% in cases of Wolf-Hirschhorn syndrome [38]. Together with the findings of the current study, these facts make us believe that genomic imprinting should be carefully considered in the heritability of either comitant or incomitant forms of strabismus.

As suggested by Strauch et al. [26], revisiting our data set using MOD score analysis allowed us to point out three new susceptibility loci on chromosomes 6, 12, and 19, which were not detected in our previous work. Even after applying adjustment values to correct for the inflation in MOD scores [12,32], the results at 6q26, 12q24.32, and 19q13.11 could at least be interpreted as suggestive evidence of linkage to comitant strabismus.

The best fitting model of inheritance, which was proposed by MOD score analysis, in the current study at the loci previously reported to be linked to comitant strabismus [6,7], showed a tendency to reduced penetrances at most of these loci, as an observation we previously reported [7]. Parikh et al. [6] suggest a semi-dominant model of inheritance in their data set and report their maximum linkage peak at chromosome 7p for a model with near complete penetrance.

Shete and Amos [17] state that if the disease etiology does not have parent-of-origin effects and linkage analysis that allows for imprinting is used, the linkage test loses power. On the other hand, they find that in case of true imprinting, linkage analysis that allows for imprinting has higher power than an analysis that does not. By the same token, Strauch et al. [26] argue that analyzing an imprinted locus by means of a dominant or recessive model is an aggravating misspecification and hence propose that for parametric linkage analysis, the maternal and paternal imprinting models should become as standard as the dominant and recessive models. By using MOD score analysis and testing for genomic imprinting in our data set, we complemented our earlier work [7,18] aiming at investigating the susceptibility loci for comitant strabismus.

In conclusion, this study suggests genomic imprinting as a possible mode of inheritance in comitant strabismus and reports three new chromosomal susceptibility loci. We propose thorough investigation of the phenomenon of parent-of-origin effect together with finer mapping at the proposed susceptibility loci as logical steps in the quest of revealing the genes underlying the inheritance of comitant strabismus.
